# Progress in Cancer

**Published:** 1901-05-25

**Authors:** 


### Progress in Cancer.

The best work published on cancer in the last few months is Adami's[1] recent paper opening the whole question of tumour formation afresh. His main endeavour is to find some ground common to all tumours, innocent or malignant; but it is difficult to bring such tumours as teratomata into line with neoplasms such as deciduoma malignum. Nevertheless he approaches and deals with the subject of the properties of the living cell in such a way as to find this common ground in a theory which is worth while to consider through the reasoning he adduces.

He starts with C. P. White's definition of a tumour, viz.:—"A tumour proper is a mass of cell tissues, or organs, resembling those normally present, but arranged atypically. It grows at the expense of the organism, without at the same time subserving any useful function."

He then classifies tumours for his purpose thus:—

A. Teratomata.

B. Blastomata.

I. In which abnormal cell relationship precedes the neoplasia by a definite interval, and apparently predisposes to the aberrant purposeless growth.

(*a*) Inherited. (*b*) Acquired. (1) Ante-natal. (2) Post-natal.

II. Tumours in which one and the same agency leads to abnormal cell relationship, and to tumour growth.

(*a*) Those arising from apparently normal and functional cells, the causative agency undetermined.

(*b*) Those arising from normal and functional cells through unknown microbic agency.

He rests upon previous work of his own in the statement that function and proliferation are the complements of each other in the cell life history. If function is required, then proliferation ceases to be active, but where stimulus acts upon a cell and function is not required, then proliferation becomes active where the cell has stored energy, and nutriment is continuously abundant.

The stimulus to increased functional activity comes from without; but the change from functional to proliferative action is not sudden, wherever the cause of the change comes from. There is marked "inertia" in the cell. Periodic activity produces an increased amount of functional activity; this is followed by a period of rest, in which energy is stored in the cell. During this quiescent period if the surroundings be altered, the tension changed or what not, then there is produced a tendency to proliferation.

This leads him then to find a common starting point for all forms of tumours in the condition "in which the cells of the part, whether functional or lying latent, upon being stimulated and as a consequence undertaking active assimilation and anabolism, cannot from their relations carry on their peculiar functions to a corresponding extent. So that the accumulated energy is instead utilised in mitotic changes and cell division rather than in the normal katabolism."

"Now," he continues, "cells and their descendants which for long periods have been subjected to certain influences whereby their properties and structures have become modified, eventually retain those properties after the influences referred to have ceased to act upon them." That is they have taken on a habit of growth which they do not lose (inertia).

He is led to this theory, that whatever the origin of the tumour proper, however it is started, what makes the tumour is the assumption of the primary cells of that tumour of the habit of growth instead of the habit of work; and according to the extent of this replacement so do we get the various grades of tumour formation from the most benign to the most malignant.

With regard to the microbic origin, microbes cannot cause all tumours (teratomata &c.), but it is quite conceivable that such microbes might continue to exist in the tumours they originated, exciting a cumulative effect. The more the cells departed from their type the greater the effect of these microbes and their products in producing a tissue of rapidly proliferative and malignant type. Yet, if this theory hold true no anti-parasitic or anti-toxin will arrest the disease. Nothing that stops short of actual destruction of the cells will do that, as they will continue their tendency to proliferate in spite of the causative agent being removed.

Following on Adami's demonstration in the tissues of cells of an embryonic type (mother cells), whose duty it is to renovate the tissues in which they are, Cooper [2] has studied the development of malignant tumours from the histogenic layers containing the mother cells in carcinomata and sarcomata.

The malignant cells depart from the normal type of histogenic cells:—

(1) In their mode of division. Karyokinesis, in his opinion, is replaced by the more abrupt and simple method of direct cell division, and frequently also the primitive methods of multiple and endogenous cell formation. He explains that apparent mitosis is simulated by nuclear degeneration or chromatolysis.

(2) In their power of movement or migration.

(3) In their power of ingesting albuminous particles.

(4) In the frequent presence of certain bodies in their interior.

(5) In their feeble resisting powers (necrosis).

(6) In their abnormal metabolism, evidenced by the production of certain toxins, which affect the blood and injure the tissues generally.

The "integrating" power of the cells is centred in the nucleus, which received its vigour, with the portion of maternal nucleus at its inception. This, possibly, being unequal to the strain ot higher development, loses irretrievably its power of producing more highly differentiated nuclei. This he offers as a suggestion of the origin of the peculiarity of cancer cells.

The cause of this loss of integrating power he does not look for in microbic or parasitic growth. He suggests that the power of integration may be faulty from the first, leading to congenital and embryonic malignant tumours, so the power may not be strong enough to resist depressing influences, instanced by traumatic sarcomata of the young. In most cases, however, there is a sufficiency of the power until age advances and the nuclear activity declines, when exceptional demands cannot be met. Long continued irritation now, for instance, will gradually exhaust the power, and hence cancers are frequent in advanced ages.

Micro-organisms may doubtless be causes of irritation, but this does not admit of their being the specific cause.

[1] Brit. Med. Jour., March 16. [2] Med. Chron., December.

